# Analysis of adolescent psychiatry hospitalization in integral beds in a city in northeast Brazil

**DOI:** 10.1192/j.eurpsy.2025.801

**Published:** 2025-08-26

**Authors:** J. L. Da Silva, F. F. D. C. Passos, J. E. D. L. Sobrinho, M. D. D. S. Leal

**Affiliations:** 1 HEBIATRIA, UPE, RECIFE; 2 FURG, Rio Grande do Sul, Brazil

## Abstract

**Introduction:**

Psychiatric hospitalization of adolescents can have biological, psychological and social impacts on these patients with psychiatric illness if not managed correctly. To achieve this, the aim is to keep the hospitalization time as short as possible and to have a clear goal of the intervention (i.e., to diagnose, monitor risks, and stabilize comorbidities). This care is important because the effectiveness of the hospitalization process and its articulations after discharge are related to the chances of these patients being readmitted.

**Objectives:**

To describe the general aspects related to the hospitalization of adolescents with psychiatric illnesses who require hospitalization in a general hospital in northeastern Brazil.

**Methods:**

This is a longitudinal study carried out in a general hospital in the state of Pernambuco, in the northeastern region of Brazil Medical records of hospitalizations that occurred between January 2018 and January 2023 were analyzed. The medical records of patients aged 12 to 17 years and 11 months were included in the study. Sociodemographic and clinical data were collected from the medical records to be submitted to statistical analysis. This study was approved by the local ethics committee (number 6.880.316).

**Results:**

Five hundred and nineteen adolescents were admitted to the general hospital under study for mental reasons throughout that time. After evaluating the data for consistency, 484 medical records were included in this study. It was observed that approximately 53.7% of hospitalized patients were male. The median age was 16 years (IQR 14-17 years). Regarding the clinical profile of hospitalizations, 39 different diagnoses of psychiatric illnesses were listed throughout the period. Following the ICD-10 classification, the three main reasons for hospitalization are: F23 - acute and transient psychotic disorders (13%); F19 - mental and behavioral disorders due to multiple drug use and use of other psychoactive substances (12.4%), and F32 - depressive episodes (11.4%). Notably, during the study period, 63 individuals were hospitalized more than one time.

**Conclusions:**

The primary mental health diagnoses of teenagers who were hospitalized to a northern Brazilian general hospital were described. From the standpoint of public health, the existence of patient readmissions highlights the deficiency of proper care provided to the adolescents after being discharged from the facility. Nevertheless, the examination of the variables associated with the hospitalization of adolescents was made difficult by the lack of uniformity and precision in medical records.

**Disclosure of Interest:**

None Declared

